# Diurnal Rhythms in Neurexins Transcripts and Inhibitory/Excitatory Synapse Scaffold Proteins in the Biological Clock

**DOI:** 10.1371/journal.pone.0037894

**Published:** 2012-05-25

**Authors:** Mika Shapiro-Reznik, Anje Jilg, Hadas Lerner, David J. Earnest, Nava Zisapel

**Affiliations:** 1 Department of Neurobiology, The George S. Wise Faculty of Life Sciences, Tel Aviv University, Tel Aviv, Israel; 2 Institute of Cellular and Molecular Anatomy, Dr. Senckenbergische Anatomie, Goethe-University, Frankfurt, Germany; 3 Department of Neuroscience and Experimental Therapeutics, Texas A&M University Health Science Center, College Station, Texas, United States of America; Harvard University, United States of America

## Abstract

The neurexin genes (NRXN1/2/3) encode two families (α and β) of highly polymorphic presynaptic proteins that are involved in excitatory/inhibitory synaptic balance. Recent studies indicate that neuronal activation and memory formation affect NRXN1/2/3α expression and alternative splicing at splice sites 3 and 4 (SS#3/SS#4). Neurons in the biological clock residing in the suprachiasmatic nuclei of the hypothalamus (SCN) act as self-sustained oscillators, generating rhythms in gene expression and electrical activity, to entrain circadian bodily rhythms to the 24 hours day/night cycles. Cell autonomous oscillations in NRXN1/2/3α expression and SS#3/SS#4 exons splicing and their links to rhythms in excitatory/inhibitory synaptic balance in the circadian clock were explored. NRXN1/2/3α expression and SS#3/SS#4 splicing, levels of neurexin-2α and the synaptic scaffolding proteins PSD-95 and gephyrin (representing excitatory and inhibitory synapses, respectively) were studied in mRNA and protein extracts obtained from SCN of C3H/J mice at different times of the 24 hours day/night cycle. Further studies explored the circadian oscillations in these components and causality relationships in immortalized rat SCN2.2 cells. Diurnal rhythms in mNRXN1α and mNRXN2α transcription, SS#3/SS#4 exon-inclusion and PSD-95 gephyrin and neurexin-2α levels were found in the SCN in vivo. No such rhythms were found with mNRXN3α. SCN2.2 cells also exhibited autonomous circadian rhythms in rNRXN1/2 expression SS#3/SS#4 exon inclusion and PSD-95, gephyrin and neurexin-2α levels. rNRXN3α and rNRXN1/2β were not expressed. Causal relationships were demonstrated, by use of specific siRNAs, between rNRXN2α SS#3 exon included transcripts and gephyrin levels in the SCN2.2 cells. These results show for the first time dynamic, cell autonomous, diurnal rhythms in expression and splicing of NRXN1/2 and subsequent effects on the expression of neurexin-2α and postsynaptic scaffolding proteins in SCN across the 24-h cycle. NRXNs gene transcripts may have a role in coupling the circadian clock to diurnal rhythms in excitatory/inhibitory synaptic balance.

## Introduction

Neurexins are neuron-specific cell-surface proteins [1], [2] that act in the vertebrate nervous system as trans-synaptic receptors and have an important role in cognition [3]. In mammals, the neurexins are encoded by three genes NRXN1, NRXN2 and NRXN3, each has two promoters that generate longer(α) and shorter(β) forms [1], [2], [3]. The neurexin genes transcripts are edited by extensive alternative splicing at five canonical sites referred to as SS#1 to SS#5 in α neurexins, two of which (SS#4, SS#5) are shared with the β neurexins [3], [4]. Neurexins’ immunoreactivity has been localized mainly to pre-synaptic nerve terminals [5] while their known ligands are found predominantly at post-synaptic sites in target cells (i.e. neuroligins, leucine-rich repeat transmembrane neuronal proteins, GABA_A_ receptors, Cbln1/Glutamate receptor delta2 and dystroglycan) or in the extracellular fluid (e.g. neurexophilins). Binding of neurexins to their ligands form trans-synaptic complexes which regulate glutamatergic and GABA-ergic transmission and subsequently the excitatory/inhibitory balance in brain networks [6]–[16].

We have recently demonstrated in one day-old rat brain neurons, that activation (by depolarization) induced a reversible, calcium-dependent splicing repression of the rNRXN2α SS#3 exon [17]. Further studies indicated that under such conditions SS#3 and SS#4 exons splicing in rNRXN3α but not rNRXN1α are also repressed [18]. Fear conditioning training in rats induced *in vivo* significant and transient repressions of hippocampal rNRXN1/2/3α, SS#4 splicing, attenuated levels of the excitatory postsynaptic scaffolding protein PSD-95 and the inhibitory postsynaptic scaffolding protein gephyrin in the hippocampus and fear memory formation [18]. Induced neuronal activity may thus affect NRXNs transcripts and subsequently synaptic fine tuning.

The circadian clock residing in the brain’s suprachiasmatic nuclei (SCNs), is responsible for the adaptation of physiological and behavioral rhythms to the external 24 h light-dark cycle and attaining proper temporal order within the organism [19]. Neurons in the SCN act as self-sustained oscillators that generate circadian rhythms in gene expression and electrical firing bursts to encode time of day, with high firing rates during the day and lower rates at night [20]. The core molecular clockwork in SCN neurons is based on autoregulatory feedback loops of transcriptional activators (CLOCK/NPAS2 and BMAL1) and inhibitors (PER1–2 and CRY1–2) [21]. The mechanisms by which the core clock produces synchronized rhythms in neural firing and gene expression are postulated to involve intracellular calcium, a second messenger that regulates many cellular processes [22]. In principle, neuronal firing bursts may trigger synaptic remodeling [23], and as such may be pivotal for the communication of the day/night information to the brain and body. Indeed there are several publications showing synaptic plasticity in SCN [24]–[31]. How the expression of genes encoding proteins that are relevant to synaptic transmission is changing across the circadian cycle is largely unknown.

Because the neurons of the circadian clock display autonomous rhythms in activity we hypothesized that they would also display autonomous oscillations in neurexins SS#3/SS#4 exons splicing and subsequently impinge on excitatory/inhibitory synaptic balance. In the present study we have thus explored circadian patterns in NRXN1/2/3α expression and SS#3/SS#4 exons splicing in the mouse SCN *in vivo*. The variations in PSD-95 and gephyrin levels were assessed as markers of excitatory/inhibitory synaptic balance.

To further explore the relationship between rhythms in NRXNs expression and SS#3/SS#4 exons splicing and the synaptic scaffold proteins we used immortalized rat SCN2.2 neurons *in vitro* [32]. These cells retain most biochemical and functional properties of the clock with internal periodicity of ∼24 hours [33]. SCN2.2 cells were used to provide homogenous neuronal-derived cell population and facilitate plasmid transfection experiments. We show here that these cells show circadian variations in rNRXN1/2 expression and SS#3/SS#4 exons splicing and in PSD-95 and gephyrin levels and intracellular localization. Finally, we used siRNAs in SCN 2.2 cells to down regulate specific neurexins transcripts for establishing their role of in the circadian rhythms in synaptic scaffold proteins.

## Results

### Circadian Rhythms in NRXNs Transcripts in the C3H/J Mouse SCN

**Figure 1:**
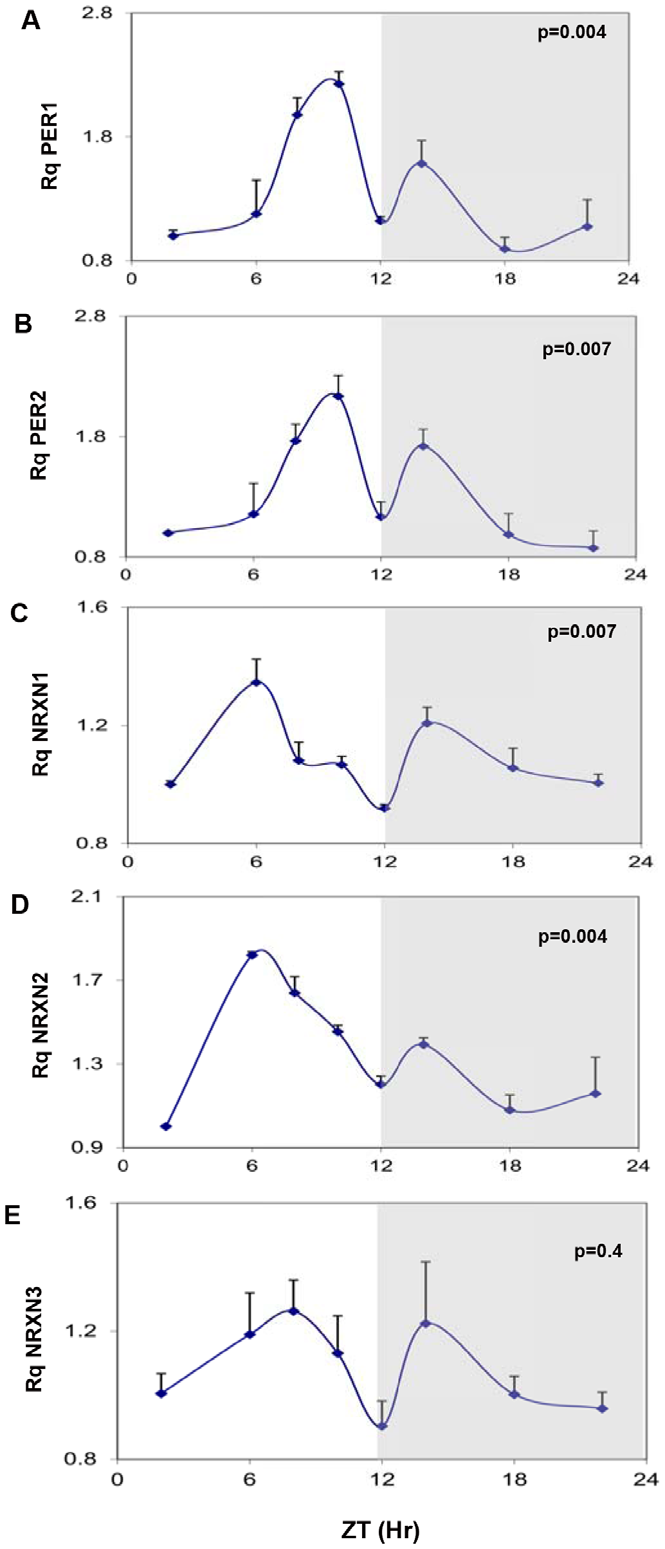
Diurnal changes in mPer1 mPer2 and mNRXN1/2/3α mRNA levels in the mouse SCN. C3H/J mice kept at 12:12 hours light:dark schedules were sacrificed at different times after light onset (Lights on at time = 0 (ZT0)). mRNA was extracted from the SCN and subjected to real-time PCR. The amount of (A) mPer1, (B) mPer2, (C) Total mNRXN1α, (D) Total mNRXN2α, and (E) Total mNRXN3α transcripts per GAPDH are presented. Values are expressed as Mean +SEM. (N = 3 animals in each time point assessed in quadruplicates). Significance of the rhythm (ANOVA) is presented on each panel. The grey bars indicate the dark phases.

The mRNA levels of the core clock genes mPer1, mPer2 showed robust rhythms in the mouse SCN (Fig. 1A and B). In agreement with previous findings in animals entrained to 12:12 hours light-dark cycles [34], two synchronous peaks of mPer1 and mPer2 transcripts were demonstrated at 2 hours before (major peak) and 2 hours after (minor peak) the beginning of the dark phase (ZT10 and ZT14) (Fig. 1A and B). The levels of total mNRXN1α and mNRXN2α transcripts also varied significantly over the 24-hour cycle (Fig. 1C and D). NRXN1α transcript levels also displayed 2 peaks (a major peak at ZT6 and a minor at ZT14), whereas NRXN2α displayed a main peak at ZT6 (Fig. 1C and D). There were no significant rhythms in mNRXN3 α transcripts in the SCN (Fig. 1E).

**Figure 2:**
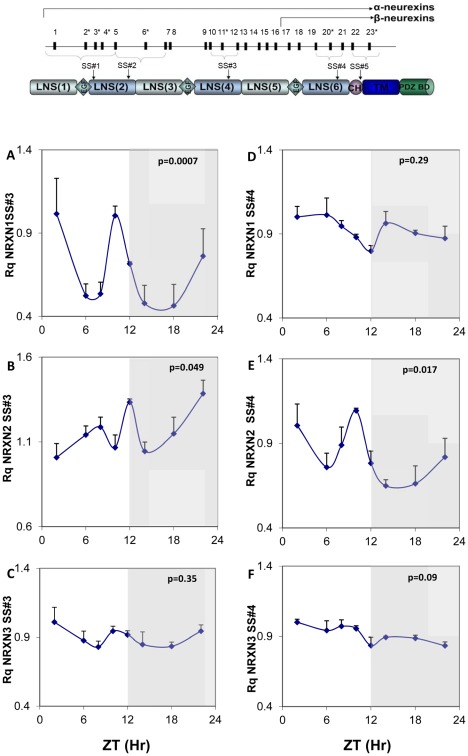
Diurnal changes in mNRXN1/2/3α SS#3/SS#4 exons splicing in the mouse SCN. Upper panel: Neurexin genes structure and splice sites. Laminin, neurexin, sex-hormone-binding protein (LNS), epidermal growth-factor)-like (EGF), transmembrane (TM) domains, highly glycosylated region (CH), and PDZ-domain-binding site (PDZ BD). The position of each of five alternative splicing sites is indicated (SS#1–SS#5). Exons are identified by numbers; asterisks mark exons subject to alternative splicing. Lower panels: C3H/J mice kept at 12:12 hours light:dark schedules were sacrificed at different times after light onset (Lights on at time = 0 (ZT0)). mRNA was extracted from the SCN and subjected to real-time PCR. The amount of SS#3 exon included transcripts of (A) mNRXN1α, (B) mNRXN2α, (C) mNRXN3α, and of SS#4 exon included transcripts of (D) mNRXN1α, (E) mNRXN2α, (F) mNRXN3α transcripts are expressed per total transcripts of the relevant mNRXN1/2/3α. Values are expressed as Mean +SEM (N = 3 animals in each time point assessed in quadruplicates). Significance of the rhythm (ANOVA) is presented on each panel. The grey bars indicate the dark phases.

Fig. 2 shows the structure of the NRXNs and circadian rhythms in mNRXN1/2/3α SS#3/SS#4 exons splicing (amount of exon included per total transcript of a particular NRXN) in the SCN. Because of 1 exon difference between NRXN1 and NRXN2, SS#3 alternative exons are numbered E12(NRXN1) and E11(NRXN2) and SS#4 alternative exons are numbered E21 (NRXN1) and E20(NRXN2). We observed significant diurnal variations in alternative exon inclusion in mNRXN1α and mNRXN2α SS#3 (E12 and E11, respectively) and mNRXN2α SS#4 (E20) transcripts (Fig. 2A, B and E). mNRXN1α SS#3 and mNRXN2α SS#4 splicing displayed two synchronous peaks each (ZT2 and ZT10) (Fig. 2A and E) and mNRXN2α SS#3 exon-included transcript displayed a main peak in the dark phase (ZT22) (Fig. 2B). These peaks did not coincide with the peak expression of the total transcripts of these genes in the SCN (Fig. 1). There were no significant rhythms in mNRXN3α SS#3 and SS#4 or mNRXN1α SS#4 splicing in the SCN (Fig. 2 C, D and F).

**Figure 3:**
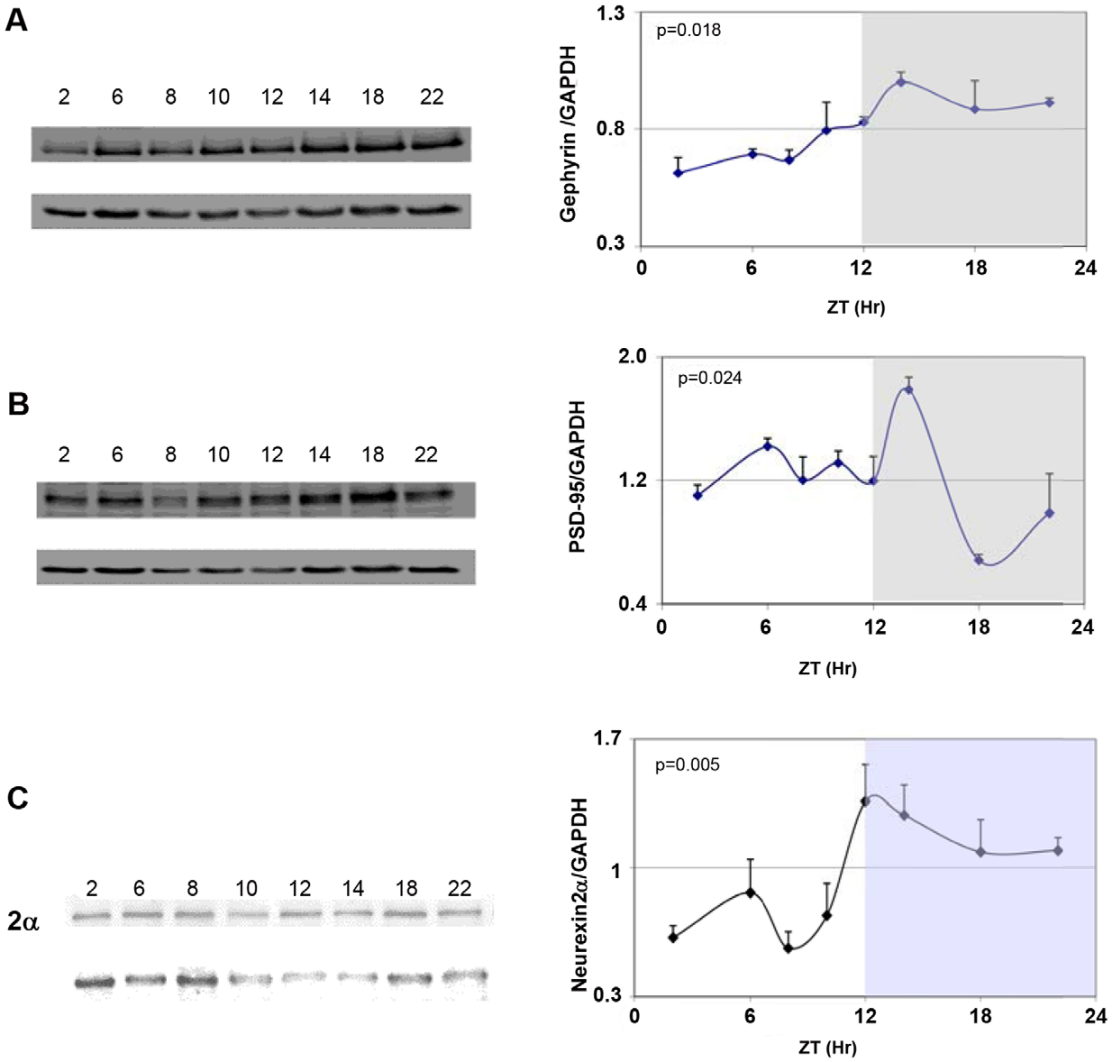
Diurnal rhythms in synaptic scaffold proteins and neurexin 2α in the mouse SCN. C3H/J mice kept at 12:12hours light:dark schedules were sacrificed at different times after light onset (Lights on at time = 0 (ZT0)). Proteins were extracted from the SCN and analyzed by immunoblotting. Representative blots of A) Gephyrin B) PSD-95 and C) neurexin2α and the corresponding GAPDH at various times after lights-on (ZT) are shown in the left panels. Quantifications of PSD-95, gephyrin and neurexin 2α levels relative to GAPDH are presented in the right panel. Values are expressed as Mean +SEM levels of respective protein relative to GAPDH (N = 3 animals in each time point). ZT represents hours from lights-on. The gray shaded areas indicate the dark phase. Significance of the rhythm (ANOVA) is presented on each panel.

Because neurexins impinge on excitatory/inhibitory synaptic balance we looked at diurnal variations in the related scaffold proteins. Figure 3 depicts the levels of gephyrin PSD-95 and neurexin 2α protein levels in the mouse SCN at different time points within a 24 h-cycle. Neurexin-1α levels were not assessed due to non-availability of specific antibodies. Gephyrin levels were significantly elevated during the dark period (ZT14-ZT22) and minimal during the day (Fig. 3A). The levels of PSD-95 protein rose during the day to reach peak levels at ZT14 and declined afterwards in the dark period to minimal values at ZT18 (Fig. 3B). Thus, both gephyrin and PSD-95 were at their peak levels at ZT14 but whereas PSD-95 level was low during the dark hours gephyrin levels were minimal during the light hours. The levels of neurexin 2α protein rose during the day to reach peak levels at ZT12 (namely ∼6 hours after the peak in mNRXN2α mRNA; Fig. 1D), and declined afterwards in the dark period to minimal values during the day (Fig. 3C) in a similar manner to gephyrin.

### Circadian Rhythms in rNRXN1/2/3 α mRNA Levels in the SCN2.2 Cells

**Figure 4:**
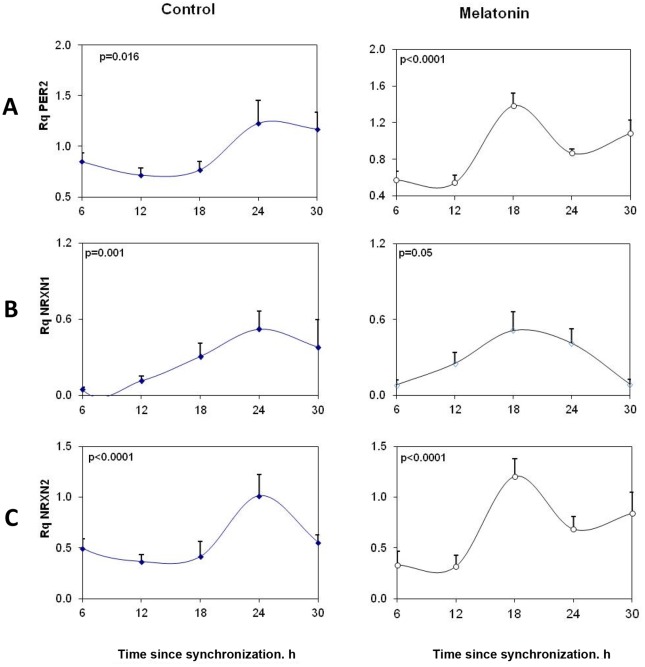
Circadian variations in rPer2, rNRXN1α and rNRXN2α expression in synchronized SCN2.2 cells and the effects of melatonin on them. Cells (6 plates each) were synchronized and incubated with vehicle (left panels) or 100 nM melatonin (right panels) for 6 hours and collected at different time points during 24 hours (6 plates each). A) rPer2; B) Total rNRXN1α C) Total rNRXN2α qPCR values are normalized vs. rGAPDH. Significance of the rhythms (ANOVA) is denoted on each panel.

The role of particular splice variants in the circadian rhythms of PSD-95 and gephyrin was further explored in vitro using SCN2.2 cells. SCN2.2 cells expressed rNRXN1α and rNRXN2α but not rNRXN3α (Fig. 4). Also, rNRXN1β and rNRXN2β were not detected in these cells. The mRNA levels of the core clock gene rPer2 and of rNRXN1/2 α in the SCN2.2 cells at different times after synchronization are presented in Figure 4. There was a significant rhythm in expression of rPer2 in the rat SCN2.2 cells, with peak at 24 hours after synchronization (t = 24). Thus, Per2 levels at t = 24 were significantly higher than at t = 12–18 hours after synchronization (P<0.001 for both). As can be seen in Figure 4, mRNA levels of rNRXN1α and rNRXN2α also varied significantly over the 24 hour cycle and displayed synchronous peaks at t = 24 hours. Thus, rNRXN1α level at t = 24 was significantly higher than at t = 6, 12 and 18 h (p<0.01 for all). rNRXN2α levels at t = 24 were significantly different than at t = 12 and 18 h (p<0.001 for both) and levels at t = 18 also differed from those at t = 6 h (p<0.01). Mesor values of rPer2, rNRXN1α and rNRXN2α transcript Rq levels per GAPDH were 0.96, 0.41 and 0.63, respectively.

Like SCN cells, immortalized cells from the rat suprachiasmatic nucleus express functional melatonin receptors [35]. To evaluate whether the changes in rNRXN1/2 expression and SS#3/SS#4 exons splicing represent clock driven rhythms we performed phase shifting experiments, using melatonin (10^−7^M at t = 0 for 6 h and incubation resumed with normal medium until harvest). In the presence of melatonin, rPer2 peak expression was advanced by ∼6 hours to t = 18 h (Fig. 4A). Peak rNRXN1α and rNRXN2α transcript levels were also advanced with melatonin from t = 24 h to t = 18. The mesor values (mean 24 h transcript levels) were not affected (0.89, 0.31 and 0.41 for rPer2, rNRXN1α and rNRXN2α transcripts, respectively) and rhythm amplitude, as represented by peak/trough rNRXN1α and rNRXN2α mRNA ratios were maintained or slightly increased under the melatonin conditions (Fig. 4B).

**Figure 5:**
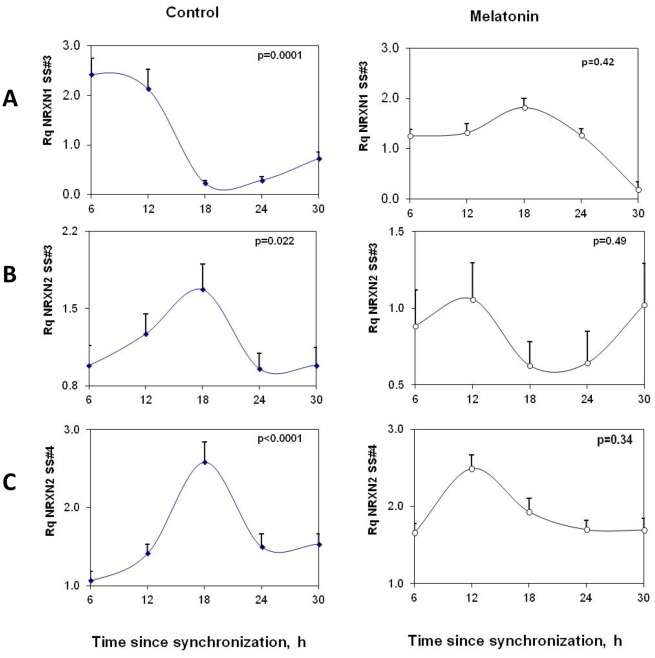
Circadian variations in rNRXN1α and rNRXN2α SS#3/SS#4 splicing in SCN2.2 cells and the effects of melatonin on them. Cells (6 plates each) were synchronized and incubated with vehicle (left panels) or 100 nM melatonin (right panels) for 6 hours and followed up afterwards for 24 hours. The amount of SS#3 exon included transcripts of (A) ) rNRXN1α, (B) rNRXN2α, and of SS#4 exon included transcripts of (C) rNRXN2α, transcripts are expressed per Total transcript of the relevant rNRXN1/2α. Significance of the rhythm (ANOVA) is denoted on each panel.

Diurnal rhythms in rNRXN2α SS#3/SS#4 exons splicing (inclusions of E11 and E20 respectively) and rNRXN1α SS#3/SS#4 exons splicing (corresponding to inclusions of E12 and E21 respectively) in the SCN2.2 cells were also assessed (Fig. 5). Significant rhythms were observed in rNRXN2α SS#3/SS#4 alternative splicing transcripts with synchronous peaks in E11/E20 exons inclusions at t = 18 h after synchronization (Fig. 5). Transcripts levels at t = 18 h were significantly higher than at t = 6 and t = 24 h and did not overlap the phase of peak expression of rNRXN2α (Fig. 4). These rhythms were attenuated with melatonin (Fig. 5). rNRXN1α SS#3 exon (E12) splicing also demonstrated a significant diurnal oscillation with peak exon included transcript at t = 12. Transcript level (per total rNRXN1α) at t = 12 was significantly higher than at t = 18 and t = 24 h after synchronization. Peak rNRXN1α SS#3 exon included transcript (per total rNRXN1α) was delayed with melatonin to t = 18 h (Fig. 5). rNRXN1α SS#4 exon (E21) included transcripts were not detected in these cells.

**Figure 6:**
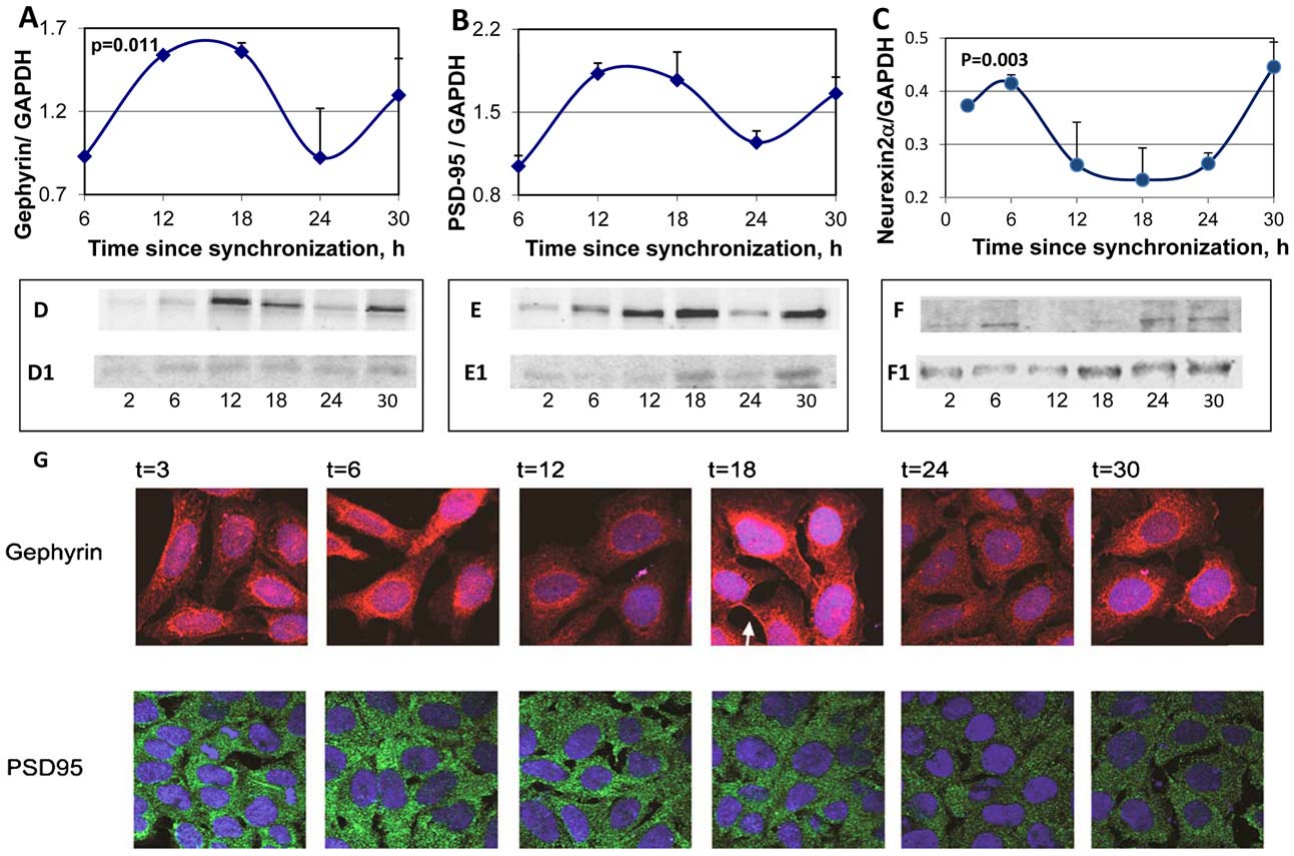
Circadian rhythms in synaptic scaffold protein and neurexin 2α levels and intracellular localization in the SCN2.2 cells. Cells (6 plates each) were synchronized and collected every 6 hours (6 plates each). Proteins were extracted from the cells and analyzed by SDS PAGE and Western blotting. Protein levels of Gephyrin (A) PSD-95 (B) and neurexin 2α (C) were quantified by immunoblots and normalized to GAPDH levels. Values are Mean +SEM; N = 3 cultures in each time point. Significance of the rhythm (ANOVA) is denoted on each panel. Representative blots of (D) Gephyrin (E) PSD-95 and (F) neurexin2α. The respective GAPDH blots from the same gels (D1, E1 and F1) at various times after synchronization are also depicted. G) SCN2.2 cells were seeded over Poly-L-Lysine coated cover slips, synchronized and fixed by 4% paraformaldehyde after different hours (T = 2–30 h). Cells were immunostained for PSD-95 and Gephyrin, and nuclei were stained with Hoechst staining. An example of membrane staining is noted by a white arrow.

The changes in levels of PSD-95 gephyrin and neurexin 2α with the circadian time were also assessed in the SCN2.2 cells (Fig. 6). Western blot analysis of the proteins indicated clear and significant circadian rhythms in the levels of the proteins, with maximal PSD-95 gephyrin levels at t = 12–18 h after synchronization. Minimal levels of both protein were observed at t = 24 h. Neurexin 2α were maximal at t = 6 h after synchronization (Fig. 6C) namely ∼6 hours after the peak in rNRXN2α mRNA (Fig. 4C).

The intracellular localization of PSD-95 and gephyrin in SCN2.2 cells at various times after synchronization, are depicted in Fig. 6C. Immunofluorescence experiments at different hours of the cycle using antibodies for PSD-95 (green) and gephyrin (red) indicated that PSD-95 was mostly associated with dense clusters in the cytoplasm at all time points. Gephyrin was mostly localized in the peri-nuclear region but showed clear membrane staining at t = 18 h and less so at other times. Unlike the Western blots that demonstrated even levels of gephyrin at t = 12–18 (Fig. 6D), gephyrin staining in the cells consistently demonstrated higher apparent intensity at t = 18 h than at t = 12 h (Fig. 6D).

**Figure 7:**
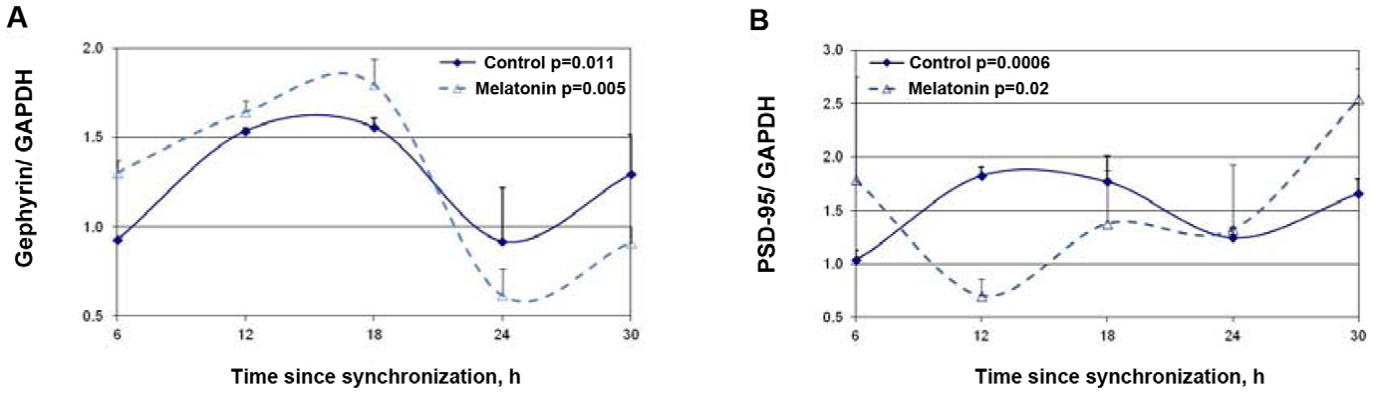
Effects of melatonin on the rhythms in synaptic scaffold protein levels in the SCN2.2 cells. Cells (6 plates each) were synchronized and incubated with vehicle (circles) or 100 nM melatonin (triangles) for 6 hours and collected every 6 hours (6 plates each). Proteins were extracted from the cells and analyzed by SDS PAGE and Western blotting. Protein levels of Gephyrin (A) and PSD-95 (B) were quantified by immunoblots and normalized to GAPDH levels. Values are Mean +SEM; N = 3 cultures in each time point. Significance of the rhythm (ANOVA) is denoted on each panel.

The effects of melatonin treatment on PSD-95 and gephyrin rhythms are depicted in Figure 7. For both, significant rhythms were still evident following 6 hours melatonin treatment (P = 0.005 for gephyrin and P = 0.02 for PSD-95) without significant changes in mesor values. Following melatonin treatment maximal PSD-95levels shifted to at 6 h (as also at 30 h) after synchronization (Fig. 7A). The phase shifting effects of melatonin treatment on gephyrin was minor, and maximal levels of Gephyrin were seen at t = 18 h after synchronization (Fig. 7B).

### Gephyrin and PSD-95 Levels are Linked to NRXN2α SS#3 Exon-including Transcripts

**Figure 8:**
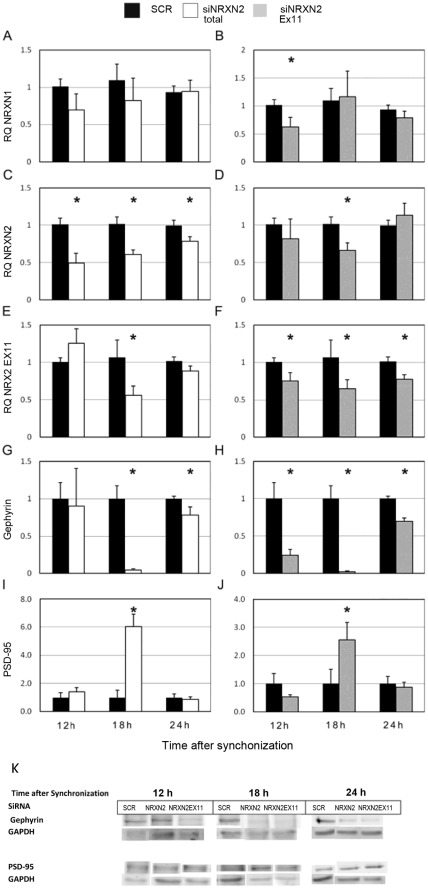
Silencing of NRXN2α and NRXN2α E11 including transcript have differential effects on gephyrin and PSD-95 levels. Total and E11 including rNRXN2α mRNA levels were suppressed using targeted siRNA sequences (siRNA NRXN2 and siRNA NRXN2 E11 respectively), a scrambled sequence (SCR) was used as a control. Cells were synchronized and then incubated with the siRNAs or SCR for 24 hours and harvested 12,18 or 24 hours afterwards. Mean (+SEM) mRNA levels of (A,B) total rNRXN1α, (C,D) total rNRXN2α, (E, F) E11 including rNRXN2α, and protein levels of (G, H) gephyrin and (I, J) PSD-95 are depicted. Significant differences from control values are marked with asterisks (* p<0.05 t-test against SCR; N = 3). (K) The respective gephyrin, PSD-95 and GAPDH blots are also depicted.

We then used siRNA mediated suppression of rNRXN1α, rNRXN2α and rNRXN2α SS#3 exon- included transcripts to explore causal relationships between rhythms in neurexin genes expression, SS#3/SS#4 alternative exons splicing and the rhythms in PSD-95 and gephyrin. The cells transfected with the respective siRNA were then harvested 12 (corresponding to time of peak levels of PSD-95 and gephyrin), 18 (corresponding to time of peak levels of NRXN2α SS#3 E11 included transcript) and 24 (corresponding to time of peak expression of rPer2 and NRXN2α) hours after synchronization. Expression levels of total rNRXN1α, rNRXN2α, and E11 included rNRXN2α transcripts, and gephyrin and PSD-95 protein levels were assessed (Fig. 8). Scrambled siRNA (SCR) was used as control in these studies.

The siRNA directed at the constitutive rNRXN2α exon E8 had no effect on rNRXN1α expression (Fig 8A) and significantly reduced NRXN2α transcripts at all 3 time points (Fig. 8C). This siRNA also reduced the amount of rNRXN2α E11 including transcripts at t = 18 (a time when this transcript prevails) significantly and to a lesser extent at t = 24 (without reaching statistical significance compared to the scrambled siRNA control) (Fig 8E). The suppression of rNRXN2α transcripts affected the levels of gephyrin and PSD at t = 18 (i.e. at which rNRXN2 E11 including transcript levels were also suppressed). Thus, a significant decrease in gephyrin and an increase in PSD-95 were noted in the rNRXN2α siRNA treated cells at t = 18 compared to scrambled siRNA control (Fig 8G and I). Also, in the rNRXN2α siRNA treated cells rNRXN2 E11 including transcripts were reduced at t = 24 (not significantly) and gephyrin levels decreased (Fig 8G) but not at t = 12 at which rNRXN2 E11 including transcripts and gephyrin were both unaffected.

The siRNA directed against rNRXN2 SS#3 exon (E11) including transcripts significantly reduced the level of rNRXN2 E11 included transcripts at all time points (Fig 8F). As expected, this siRNA also reduced the amount of rNRXN2 α transcripts at t = 18, (at which rNRXN2 SS#3 exon (E11) included transcripts prevail), but not at the other time points (Fig 8D). It also reduced rNRXN1α expression at t = 12 (at which rNRXN1 SS#3 exon (E12) included transcripts prevail, see Fig 5A) but not at the other times (Fig 8B). The down regulation of rNRXN2 SS#3 exon (E11) included transcripts led to significant diminution of gephyrin levels at all times. In addition rNRXN2 E11 siRNA caused significant upregulation of PSD-95 exclusively at t = 18 (a time at which rNRXN2α transcript levels were also reduced). Down-regulation of rNRXN1α with siRNA had no significant effects on PSD-95 or gephyrin (not shown).

To further exclude off-target effects of NRXN2α and NRXN2 E11 siRNAs on gephyrin, two additional siRNAs directed at other constitutive rNRXN2α exons (E19, E14) were tested. As expected, only those siRNAs that reduced rNRXN2 E11 transcripts and at the time they did so, also reduced gephyrin levels. Thus, the siRNA directed at the constitutive rNRXN2α exon E19, did not reduce rNRXNα2 levels at 24 h after synchronization but reduced mean(SEM) rNRXN2 SS#3 exon (E11) included transcripts levels to 0.54 (0.21) and gephyrin to 0.7 (0.1) of control values with scrambled siRNA. The siRNAs directed at the constitutiverNRXN2α exon E14 reduced rNRXN2α to 0.79(0.14) but not rNRXN2α SS#3 exon E11 -included transcripts at 18 h after synchronization and did not affect gephyrin levels significantly.

## Discussion

This study demonstrates diurnal variations in expression and SS#3/SS#4 splicing of mNRXN1α and mNRXN2α (but not NRXN3α) neurexin-2α and postsynaptic scaffolding proteins (PSD-95 and gephyrin) levels in the mouse SCN *in vivo*. Neurexin-2α protein peak appeared ∼6 hours after the mRNA peak. Rhythmic expression and SS#3/SS#4 exons splicing of rNRXN1α and rNRXN2α (NRXN3α is not expressed) was also observed in rat SCN2.2 cells *in vitro*. Being found in SCN samples from mouse and SCN-derived cells from rat, these rhythms appear to be a general rather than species- specific trait of SCN cells.

In the SCN *in vivo*, we observed traces of two different oscillations in clock control genes (mPer1 and mPer2) and in mNRXN1/2 genes, by which transcripts reached two peaks, one in the late light phase and another at the beginning of the dark phase. Such split was already demonstrated previously in mice kept in 12:12 light: dark cycles as in our experiment [34]. Some evidence could also be noted in other publications [36], [37]. The early peaks in mPer1 and mPer2 were ascribed to the posterior SCN and the later one to the anterior SCN [34] or to existence of separate oscillating cell groups in mouse suprachiasmatic nucleus that couple photoperiodicity to the onset and end of daily activity [34]. A recent paper [38] concluded that in the living SCN tissue individual cells undergo an oscillation in expression of genes and proteins as they participate in complex interactions across the circadian cycle. In this sense they are clearly ‘oscillating cells’, but their temporal behavior depends critically on their location in the SCN tissue, most probably as a reflection of intercellular communication. The precise timing of PER2 expression within individual neurons is dependent on their location within the nucleus, and that small groups of neurons within the SCN give rise to distinctive and identifiable sub-regions.

The intrinsic nature of the rhythms in rNRXN1/2α expression and SS#3/SS#4 exons splicing is demonstrated in rat immortalized SCN2.2 cells in culture and the ability of melatonin to phase shift them as it does for the core clock components (e.g. Per2) [35]. As SCN2.2 cells act as self sustained pacemakers [32] these results suggest that these rhythms are cell- autonomous. NRXN1α and NRXN2α genes do not include E- box sequences in their promoters and therefore cannot be regarded as primary clock-controlled genes [39]. The rhythms in expression of NRXN1α and NRXN2α and their alternative SS#3/SS#4 exons splicing thus reveal an additional layer of post-transcriptional regulation of clock controlled genes in mammals as recently found in plants (Arabidopsis) and flies (drosophila) [40].

The rhythm in PSD-95 in the SCN2.2 cells appears to be causally linked to the clock (as it was shifted/modified following melatonin treatment. The rhythm in gephyrin appears to be less dependent on clock phase. It was influenced by NRXN2α SS#3 exon (E11)-included transcript levels and was consistently modified following specific NRXN2α E11 siRNA treatments. With altogether 6 different siRNAs (one scrambled, one targeting rNRXN1, and four targeting rNRXN2 Exons E8, E11, E14 and E19) only those that reduced rNRXN2 E11 transcripts levels and at the times they did so, caused down regulation of gephyrin. Moreover, down regulation of rNRXN2α expression using siRNA directed against a constitutive exon (e.g. E8) resulted in suppression of gephyrin selectively at times when E11 including transcripts comprised most of the NRXN2α mRNA (t = 18 h). The rNRXN2α E11 SiRNA also reduced the levels of NRXN1α mRNA at t = 12 (a time when the SS#3 exon (E12) included transcripts prevail). However, as down regulation of rNRXN1α by specific SiRNA did not affect gephyrin, NRXN1 is probably not involved in the down regulation of gephyrin at t = 12.

Notably, the % of inhibition is calculated from the amount of transcript/protein in the siRNA treated cells to respective values in SCR treated cells at the same time after synchronization. As the amounts of total transcripts of the two neurexins as well as synaptic scaffolds proteins vary with the circadian cycle and at different phases, it is not surprising that the % inhibition at a certain time point may differ for the mRNA transcripts and protein levels. Therefore a high percentage at one time may be related to a small change in actual transcript or protein levels and vice versa. For example, at 12 hours most of the rNRXN2 is in the E11 excluded form and therefore even for an equal reduction in total NRXN2, that reduction is presumably in the E11 excluded form and would not lead to decrease in gephyrin at this hour (Fig. 8E). PSD-95 levels were not affected by changes in rNRXN2α E11 transcripts but upregulated under conditions that total rNRXN2α mRNA transcripts were suppressed. The effects on the synaptic scaffold proteins appear to be specific for NRXN2α as down-regulation of NRXN1α was not associated with significant changes in PSD-95 or gephyrin. Beside diurnal fluctuations in protein levels, gephyrin also exhibited diurnal rhythms in intracellular localization moving between the perinuclear region, the cytoplasm and plasma membrane. Considering the limitations of immunostaining technique as a quantitative method, the results obtained using these different methods are reasonably consistent. However, at t = 12 h after synchronization gephyrin immunostaining intensity appeared to be less than expected according to the western blot data. At this time gephyrin was mostly associated with the cytoplasm. The most obvious explanation for the apparent discrepancy is that the two antibodies (the one used in the immunoblotting and the one used in the immunostaining) differ in their affinities to some unspecified gephyrin isoforms. This remains to be further investigated.

This study suggests a link between the expression of certain NRXN splice variants to excitatory/inhibitory synapse scaffolds in the biological clock. Because there are currently no antibodies capable of detecting the specific variants of native neurexins (comprising ∼1000 isoforms), it was only possible to demonstrate circadian rhythms in total endogenous neurexin-2α and show that it followed peak NRXN2α transcripts by ∼6 h in both SCN in vivo and in the SCN2.2 cells in vitro. Nevertheless, the data in our study demonstrates that the clock controlled changes in certain endogenous NRXNs transcripts are eventually translated into changes in synaptic proteins. The molecular mechanisms linking the circadian clock to the control of RNA processing is compatible with emerging evidence suggesting that regulation at the RNA level strongly impacts the cellular circadian transcriptome and proteome as well as the oscillator mechanism itself [41].

What our study also shows is that the cross-talk between Nrxn2α E11 included transcripts and the inhibitory synapse scaffold protein Gephyrin are fast enough to be involved in synaptic plasticity over the 24 hours period. Emerging evidence suggests that the entire synaptic structure, including scaffolding proteins and vesicles, is highly dynamic during both development and periods of plasticity. Indeed, SCN oscillations are associated with plastic changes in configuration of the SCN cell network [28], [29], [31], [42], [43]. Furthermore, it appears that some of these changes involve inhibitory synapses. [44], [45]. Dynamics of GABAergic synaptic components have been studied previously over milliseconds to minutes, revealing mobility of postsynaptic scaffolds and receptors. [46], [47], [48]. Recent studies in both vertebrate and invertebrate models have shown time-of-day effects on neurophysiology and memory formation, and have revealed a possible role for cycling molecules in memory persistence (reviewed in [49]). Together, these studies suggest that common mechanisms underlie circadian rhythmicity and long-term memory formation. The rhythmic expression and SS#3/SS#4 exons splicing of NRXNs and the effects of NRXNs on the expression of postsynaptic scaffolding proteins in SCN neurons across the light-dark cycle may link the circadian clock to diurnal oscillations in balance of excitatory/inhibitory neurotransmission.

## Materials and Methods

### Animals and Sample Acquisition

All animal experiments were conducted in accordance with a protocol approved by the Policy on the Use of Animals in Neuroscience Research, the Policy on Ethics, by the Society for Neuroscience, and were consistent with Federal Guidelines and the European Communities Council Directive (89/609/EEC). Animal and sample acquisition was performed as described previously [50]. Male C3H/J mice (Charles River, Sulzbach, Germany) aged 8–12 weeks were used in all experiments. At least 2 weeks prior to experiments, animals were housed under a standard light-dark (LD) regimen, with 12-h light (250 lux; light on designated as Zeitgeber time [ZT0] and 12-h darkness (dim red light <10 lux, >680 nm), with constant room temperature, and food and water available *ad libitum*. Tissue sampling was carried out at ZT2, 6, 8, 10, 12, 14, 18, and 22 (three animals per time point). After decapitation of animals at indicated time points, the brain was removed. The isolation of the SCN essentially followed published procedures [19], [51]. Briefly, after brain removal, 500 µm thick slices of the suprachiasmatic region were prepared with a manual tissue chopper. When the desired level of SCN has been reached (the SCN at this point appears as more defined, round or almond shaped structures) sections containing the SCN were cut from the slices under a binocularly microscope using scalpels and surrounding tissue was carefully removed. The isolated SCN tissues were immediately frozen on dry ice. Subsequently, RNA and protein extraction was performed using the NucleoSpin**®** RNA/Protein kit according to manufacturer’s protocol. (Macherey-Nagel, Düren, Germany). Verso™ RT-PCR Kit (Thermo scientific, ABgene, Epsom, UK) was used for cDNA synthesis, from 250 ng of total RNA using Oligo(dT) primer, according to manufacturer’s protocol.

### SCN2.2 Cell Culture and Sample Acquisition

SCN2.2 Immortalized rat SCN cells, obtained from Dr. David J Earnest [32] were plated onto 6-well plastic tissue culture plates (3×10^5^/well) and grown for 48 h to confluence in minimal essential medium (MEM) supplemented with 10% fetal bovine serum (FBS), 4mM L-Glutamine, 4 µg/mL glucose (Merck, Germany), penicillin (100U/mL) and streptomycin (0.1 mg/mL) (all from Biological Industries, Beth Haemek, Israel) at 37^o^C in humidified atmosphere with 5% CO_2_. Synchronization was performed essentially as described [52]. Growth medium was replaced with medium containing 5% FBS for at least 8 hr prior to the synchronizing pulse. The medium was then removed, and cells were rinsed with calcium-magnesium free (CMF) Buffer (Dulbecco’s PBS (1X) w/o Ca^2+^ or Mg^+^ (Sigma)) containing 2 µg/mL glucose, and incubated for 1 hr in serum free medium. The serum-free MEM medium was then replaced with growth medium and cells were kept in this medium until harvested at 6 hours intervals over 30 hours. Samples were subjected to RNA extraction using MasterPure™ Kit (EPICENTRE, Madison, USA), according to manufacturer’s protocol. Verso™ RT-PCR Kit (ABgene Nucleic Acid Amplification, Epson, UK) was used for cDNA synthesis, from 1 µg of total RNA using Oligo (dT) primer according to manufacturer’s protocol. Synchrony was checked by measuring circadian rhythms in *RPer2* mRNA expression using real time PCR. Proteins extraction was performed using Radio Immuno Precipitation Assay buffer (RIPA lysis buffer; 150 mM sodium chloride, 1% NP-40, 0.5% sodium deoxycholate, 0.1% SDS (sodium dodecyl sulphate) 50 mM Tris, pH 8.0) with Protease Inhibitor Cocktail (Sigma-Aldrich, MO, USA). Cell suspension was maintained for 20min in ice, following centrifugation at 4°C for 10min at maximum speed. Supernatant was removed to a new cold tube, and proteins amount was measured spectrophotometrically with Bradford reagent at 595 nm (Bio-Rad laboratories, CA, USA).

### RNA Interference

SCN2.2 cells were plated into 6-well plates, and transfected 24 h later with 40nM of siRNA duplexes specific for rNRXN1 and for rNRXN2 exons E8, E11, E18, E19, and scrambled negative control siRNAs as a mock transfection (IDT technologies) with 8mL of INTERFERin transfection reagent (Polyplus-transfection Inc., NY, USA) according to the manufacturer’s instructions (Mirus Bio, Madison, USA). Cells were synchronized 48 hr after transfection and sampled after the indicated period of time in hexaplicates. Samples were harvested for RNA and protein extraction RT-PCR and real-time PCR experiments.

### Real Time PCR

Real-time PCR analysis was performed using ABI PRISM 7300 (Applied Biosystems, California, US) and KAPA SYBR® FAST ABI Prism® 2X qPCR Master Mix (Kapa Biosystems, Boston, US), according to the instruction of the manufacturer. Each PCR reaction mixture contained 1 µL of cDNA template mix. The primers used for real time PCR analysis are depicted in Table 1. Gene expression values for the various NRXN1/2/3α RNA and RNA splice variants were calculated based on the comparative threshold cycle (Ct) method [53]. Ct data for a specific NRXN isoform, total NRXN mRNA and the housekeeping gene GAPDH mRNA in each sample were used to create ΔCt values for total NRXN in sample (Ct _total NRXN_ – Ct _GAPDH_) and specific isoform transcripts (Ct _specific NRXN isoform_ – Ct _total NRXN_). Thereafter, ΔΔCt values were calculated by subtracting the ΔCt of the untreated control sample from the Ct value of treated sample. In circadian rhythm assessments (wherein all samples at various time points were under the same treatment) ΔΔCt values were calculated by subtracting the ΔCt at t = 0 from the Ct values of other time points with the same treatment. Rq  = 2^(–ΔΔCt)^.

**Table 1:** List of the primers that were used for qPCR.

Gene Name	Primer Sequence
GAPDH	**Fw 5' GACAACTTTGGCATCGTGGA**
	**Rv 5' ATGCAGGGATGATGTTCTGG**
mPer1	**Fw 5' CAGGCTAACCAGGAATATTACC**
	**Rv 5' TGTCAGGAAGGACACAGCC**
mPer2	**Fw 5'CTGGCTTCACCATGCCTGTT**
	**Rv 5' AAGGCCTGAGGCAGGTTTG**
NRXN1 total	**Fw 5' CATTGCAAGCCTACACTTCTATGC**
	**Rv 5' AGGTTAGCACCATTTCCCAAGTC**
NRXN1 SS#3	**Fw 5' CAGGCTATCTCGGCAGG**
	**Rv 5' GCTTGTTGATCATCCACG**
NRXN1 SS#4	**Fw 5' GCAATGGGATGGCTTCAGCTG**
	**Rv 5' CGCTGTCTAGCAATCGCCAG**
NRXN2 total	**Fw 5' TCCAGGGACCCAGGCAAC**
	**Rv 5' CTTGCTCAGGCCACCGATG**
NRXN2 SS#3	**Fw 5' GTCCCTGCGATTCATGTCCC**
	**Rv 5' CAGCCGACGCGCAGG**
NRXN2 SS#4	**Fw 5' GCCCTGTCTGCAATGAC**
	**Rv 5' GCGCTCGTTATCAAAGTTC**
NRXN3 total	**Fw 5' ACTCGGGACAACAGTAATACCCAC**
	**Rv 5' CTGGGCTAAGCCAGCCATATAG**
NRXN3 SS#3	**Fw5' CTGTATCAGGATAAACTGTAACTCC**
	**Rv 5' CCACATCATCGTCCACTGTT**
NRXN3 SS#4	**Fw 5' CTGGAAACCAGTGCAATGACC**
	**Rv 5' GGCGTTCATTATCAGTGTTGC**

### Proteins Extraction and Analyses

#### Tissue protein samples

(10–30 µg) were subjected to sodium-dodecyl sulfate polyacrylamide gel electrophoresis (SDS-PAGE) using 10% acrylamide gels (Bio-rad, California, US) followed by electroblotting onto PVDF membranes (Millipore, Massachusetts, USA). Blots were blocked with blocking buffer (5% non-fat dry milk in PBS with 0.1% Tween 20 (PBST)) for 1 hour at room temperature. Blots were incubated with the relevant primary antibody (PSD-95 ab18258 1:1000, gephyrin ab32206 1:1000, neurexin2α ab34245 1:500 and GAPDH ab8245 1:1000, all from ABCAM, Mass. USA) in PBS overnight at 4°C.

#### Cell protein samples

Aliquots (40 µg) were subjected to SDS-PAGE on 10% acrylamide gels (Bio-rad, California, US) as described above. Blots were washed and subsequently incubated with secondary antibodies IRDye® 800CW Conjugated Goat Anti-Rabbit IgG or IRDye® 680CW Conjugated Goat Anti-Mouse IgG (LI-COR Biosciences, Nebraska, USA) diluted 1∶10000. Membranes were scanned and analyzed using LI-COR Odyssey (LI-COR Biosciences, Nebraska, USA).

### Immunofluorescence

SCN2.2 cells (3×10^5^) were plated onto Poly-L-Lysine coated (Sigma-Aldrich, MO, USA) cover slips, immersed in 6-well plates and synchronized as described above. Cells were washed with serum-free Dulbecco's modified Eagle's medium and fixed with 4% paraformaldehyde in phosphate-buffered saline for 45 min at room temperature. The cells were then washed three times with cold Hanks' balanced salt solution (HBSS) (Biological Industries, Beit Haemek), supplemented with 20 mM Hepes (pH 7.4) and 2% BSA (Hepes and BSA purchased from Sigma), and permeablized with 0.25% Triton X-100 for 5 min. Fixed cells were washed again and incubated with normal goat γ globulin 200 µg/mL, (Jackson ImmunoResearch Laboratories, PA, USA), 30 min at room temperature, followed by incubation in HBSS/Hepes/BSA with primary mouse anti-PSD-95 (PSD-95 antibody [6G6–1C9] (ab2723) Abcam, Massachusetts,USA), rabbit anti-gephyrin (ALX-210–400 Enzo Life Sciences, PA,USA) for 1 h. Secondary labeling was then performed using Alexa flour® 546 Donkey anti-rabbit (Molecular Probes, Invitrogen, Oregon, USA) and cy2-goat anti-mouse IgG (Jackson ImmunoResearch Laboratories, PA, USA) for 30 min at room temperate. Cells were washed with HBSS/Hepes, stained with Hoechst dye (Sigma-Aldrich, MO, USA) and mounted with Prolong gold Antifade reagent (Molecular Probes, Invitrogen, CA, USA). Fluorescence Images were digitalized using a LSM 510 Laser Scanning Microscope (Zeiss, USA).

### Statistical Analyses

The mean (SD) values of experimental and control groups were compared using Student’s t tests. Differences were considered significant if P<0.05. Circadian rhythms were evaluated by one-way ANOVA of data from samples collected at different times of the day (P<0.05). One way (time of day) ANOVA was used to establish the presence circadian rhythms [54]. This method assesses whether the variance between time points is significantly greater than the random variation within them. The analysis requires several readings to be taken at each of a group of times within the day; the times of assessment do not have to be evenly spaced. Strength of the ANOVA method is that shape of the rhythm does not affect the statistical outcome [54]. Cosinor best fit method that is sometimes used for the analysis of circadian rhythms was not chosen because for most of the observed rhythms the data span is not sinusoidal in shape.

## References

[1] Ushkaryov YA, Petrenko AG, Geppert M, Sudhof TC (1992). Neurexins: synaptic cell surface proteins related to the alpha-latrotoxin receptor and laminin.. Science.

[2] Ushkaryov YA, Hata Y, Ichtchenko K, Moomaw C, Afendis S (1994). Conserved domain structure of beta-neurexins. Unusual cleaved signal sequences in receptor-like neuronal cell-surface proteins.. J Biol Chem.

[3] Missler M, Sudhof TC (1998). Neurexins: three genes and 1001 products.. Trends Genet.

[4] Ullrich B, Ushkaryov YA, Sudhof TC (1995). Cartography of neurexins: more than 1000 isoforms generated by alternative splicing and expressed in distinct subsets of neurons.. Neuron.

[5] Dean C, Scholl FG, Choih J, DeMaria S, Berger J (2003). Neurexin mediates the assembly of presynaptic terminals.. Nat Neurosci.

[6] Kang Y, Zhang X, Dobie F, Wu H, Craig AM (2008). Induction of GABAergic postsynaptic differentiation by alpha-neurexins.. J Biol Chem.

[7] Graf ER, Zhang X, Jin SX, Linhoff MW, Craig AM (2004). Neurexins induce differentiation of GABA and glutamate postsynaptic specializations via neuroligins.. Cell.

[8] Scheiffele P, Fan J, Choih J, Fetter R, Serafini T (2000). Neuroligin expressed in nonneuronal cells triggers presynaptic development in contacting axons.. Cell.

[9] Kattenstroth G, Tantalaki E, Sudhof TC, Gottmann K, Missler M (2004). Postsynaptic N-methyl-D-aspartate receptor function requires alpha-neurexins.. Proc Natl Acad Sci U S A.

[10] Missler M, Zhang W, Rohlmann A, Kattenstroth G, Hammer RE (2003). Alpha-neurexins couple Ca^2+^ channels to synaptic vesicle exocytosis.. Nature.

[11] Zhang W, Rohlmann A, Sargsyan V, Aramuni G, Hammer RE (2005). Extracellular domains of alpha-neurexins participate in regulating synaptic transmission by selectively affecting N- and P/Q-type Ca^2+^ channels.. J Neurosci.

[12] Kornau HC, Schenker LT, Kennedy MB, Seeburg PH (1995). Domain interaction between NMDA receptor subunits and the postsynaptic density protein PSD-95.. Science.

[13] O’Brien RJ, Lau LF, Huganir RL (1998). Molecular mechanisms of glutamate receptor clustering at excitatory synapses.. Curr Opin Neurobiol.

[14] El-Husseini AE, Schnell E, Chetkovich DM, Nicoll RA, Bredt DS (2000). PSD-95 involvement in maturation of excitatory synapses.. Science.

[15] Ehrlich I, Malinow R (2004). Postsynaptic density 95 controls AMPA receptor incorporation during long-term potentiation and experience-driven synaptic plasticity.. J Neurosci.

[16] Levinson JN, El-Husseini A (2005). Building excitatory and inhibitory synapses: balancing neuroligin partnerships.. Neuron.

[17] Rozic-Kotliroff G, Zisapel N (2007). Ca^2+^ -dependent splicing of neurexin IIalpha.. Biochem Biophys Res Commun.

[18] Rozic G, Lupowitz Z, Piontkewitz Y, Zisapel N (2011). Dynamic changes in neurexins’ alternative splicing: role of rho-associated protein kinases and relevance to memory formation.. PLoS One.

[19] Yamazaki S, Numano R, Abe M, Hida A, Takahashi R (2000). Resetting central and peripheral circadian oscillators in transgenic rats.. Science.

[20] Liu C, Weaver DR, Strogatz SH, Reppert SM (1997). Cellular construction of a circadian clock: period determination in the suprachiasmatic nuclei.. Cell.

[21] Reppert SM, Weaver DR (2002). Coordination of circadian timing in mammals.. Nature.

[22] Lundkvist GB, Kwak Y, Davis EK, Tei H, Block GD (2005). A calcium flux is required for circadian rhythm generation in mammalian pacemaker neurons.. J Neurosci.

[23] O’Brien RJ, Kamboj S, Ehlers MD, Rosen KR, Fischbach GD (1998). Activity-dependent modulation of synaptic AMPA receptor accumulation.. Neuron.

[24] Guldner FH, Ingham CA (1979). Plasticity in synaptic appositions of optic nerve afferents under different lighting conditions.. Neuroscience letters.

[25] Nishikawa Y, Shibata S, Watanabe S (1995). Circadian changes in long-term potentiation of rat suprachiasmatic field potentials elicited by optic nerve stimulation in vitro.. Brain research.

[26] Fukunaga K, Horikawa K, Shibata S, Takeuchi Y, Miyamoto E (2002). Ca^2+^/calmodulin-dependent protein kinase II-dependent long-term potentiation in the rat suprachiasmatic nucleus and its inhibition by melatonin.. Journal of neuroscience research.

[27] Gompf HS, Allen CN (2004). GABAergic synapses of the suprachiasmatic nucleus exhibit a diurnal rhythm of short-term synaptic plasticity.. The European journal of neuroscience.

[28] Kretschmannova K, Svobodova I, Balik A, Mazna P, Zemkova H (2005). Circadian rhythmicity in AVP secretion and GABAergic synaptic transmission in the rat suprachiasmatic nucleus.. Ann N Y Acad Sci.

[29] Kalsbeek A, Foppen E, Schalij I, Van Heijningen C, van der Vliet J (2008). Circadian control of the daily plasma glucose rhythm: an interplay of GABA and glutamate.. PLoS One.

[30] Moldavan MG, Allen CN (2010). Retinohypothalamic tract synapses in the rat suprachiasmatic nucleus demonstrate short-term synaptic plasticity.. Journal of neurophysiology.

[31] Girardet C, Blanchard MP, Ferracci G, Leveque C, Moreno M (2010). Daily changes in synaptic innervation of VIP neurons in the rat suprachiasmatic nucleus: contribution of glutamatergic afferents.. Eur J Neurosci.

[32] Earnest DJ, Liang FQ, DiGiorgio S, Gallagher M, Harvey B (1999). Establishment and characterization of adenoviral E1A immortalized cell lines derived from the rat suprachiasmatic nucleus.. J Neurobiol.

[33] Womac AD, Burkeen JF, Neuendorff N, Earnest DJ, Zoran MJ (2009). Circadian rhythms of extracellular ATP accumulation in suprachiasmatic nucleus cells and cultured astrocytes.. The European journal of neuroscience.

[34] Inagaki N, Honma S, Ono D, Tanahashi Y, Honma K (2007). Separate oscillating cell groups in mouse suprachiasmatic nucleus couple photoperiodically to the onset and end of daily activity.. Proc Natl Acad Sci U S A.

[35] Rivera-Bermudez MA, Masana MI, Brown GM, Earnest DJ, Dubocovich ML (2004). Immortalized cells from the rat suprachiasmatic nucleus express functional melatonin receptors.. Brain research.

[36] Field MD, Maywood ES, O’Brien JA, Weaver DR, Reppert SM (2000). Analysis of clock proteins in mouse SCN demonstrates phylogenetic divergence of the circadian clockwork and resetting mechanisms.. Neuron.

[37] Hastings MH, Field MD, Maywood ES, Weaver DR, Reppert SM (1999). Differential regulation of mPER1 and mTIM proteins in the mouse suprachiasmatic nuclei: new insights into a core clock mechanism.. The Journal of neuroscience : the official journal of the Society for Neuroscience.

[38] Foley NC, Tong TY, Foley D, Lesauter J, Welsh DK (2011). Characterization of orderly spatiotemporal patterns of clock gene activation in mammalian suprachiasmatic nucleus.. The European journal of neuroscience.

[39] Gekakis N, Staknis D, Nguyen HB, Davis FC, Wilsbacher LD (1998). Role of the CLOCK protein in the mammalian circadian mechanism.. Science.

[40] Petrillo E, Sanchez SE, Kornblihtt AR, Yanovsky MJ (2011). Alternative splicing adds a new loop to the circadian clock.. Communicative & integrative biology.

[41] Staiger D, Koster T (2011). Spotlight on post-transcriptional control in the circadian system.. Cell Mol Life Sci.

[42] Becquet D, Girardet C, Guillaumond F, Francois-Bellan AM, Bosler O (2008). Ultrastructural plasticity in the rat suprachiasmatic nucleus. Possible involvement in clock entrainment.. Glia.

[43] Meijer JH, Michel S, Vanderleest HT, Rohling JH (2010). Daily and seasonal adaptation of the circadian clock requires plasticity of the SCN neuronal network.. Eur J Neurosci.

[44] Moore RY, Speh JC (1993). GABA is the principal neurotransmitter of the circadian system.. Neurosci Lett.

[45] Decavel C, Van den Pol AN (1990). GABA: a dominant neurotransmitter in the hypothalamus.. J Comp Neurol.

[46] Goda Y, Davis GW (2003). Mechanisms of synapse assembly and disassembly.. Neuron.

[47] McAllister AK (2007). Dynamic aspects of CNS synapse formation.. Annual review of neuroscience.

[48] Dobie FA, Craig AM (2011). Inhibitory synapse dynamics: coordinated presynaptic and postsynaptic mobility and the major contribution of recycled vesicles to new synapse formation.. The Journal of neuroscience: the official journal of the Society for Neuroscience.

[49] Gerstner JR, Yin JC (2010). Circadian rhythms and memory formation.. Nature reviews Neuroscience.

[50] Jilg A, Lesny S, Peruzki N, Schwegler H, Selbach O (2010). Temporal dynamics of mouse hippocampal clock gene expression support memory processing.. Hippocampus.

[51] Gillette MU (1986). The suprachiasmatic nuclei: circadian phase-shifts induced at the time of hypothalamic slice preparation are preserved in vitro.. Brain research.

[52] Hurst WJ, Mitchell JW, Gillette MU (2002). Synchronization and phase-resetting by glutamate of an immortalized SCN cell line.. Biochemical and biophysical research communications.

[53] Livak KJ, Schmittgen TD (2001). Analysis of relative gene expression data using real-time quantitative PCR and the 2(-Delta Delta C(T)) Method.. Methods.

[54] Minors DS, Waterhouse JM (1988). Mathematical and statistical analysis of circadian rhythms.. Psychoneuroendocrinology.

